# NF-κB p65 Attenuates Cardiomyocyte PGC-1α Expression in Hypoxia

**DOI:** 10.3390/cells11142193

**Published:** 2022-07-13

**Authors:** Inna Rabinovich-Nikitin, Alexandra Blant, Rimpy Dhingra, Lorrie A. Kirshenbaum, Michael P. Czubryt

**Affiliations:** 1 Institute of Cardiovascular Sciences, St. Boniface Hospital Albrechtsen Research Centre, Winnipeg, MB R2H 2A6, Canada; irabinovich-nikitin@sbrc.ca (I.R.-N.); rdhingra@sbrc.ca (R.D.); 2 Department of Physiology and Pathophysiology, Rady Faculty of Health Sciences, University of Manitoba, Winnipeg, MB R3E 0J9, Canada; 3 Department of Biochemistry and Medical Genetics, Rady Faculty of Health Sciences, University of Manitoba, Winnipeg, MB R3E 0J9, Canada; umblant@myumanitoba.ca

**Keywords:** NF-κB, hypoxia, PGC-1α, mitochondria, gene transcription, cardiac myocytes

## Abstract

Hypoxia exerts broad effects on cardiomyocyte function and viability, ranging from altered metabolism and mitochondrial physiology to apoptotic or necrotic cell death. The transcriptional coactivator peroxisome proliferator-activated receptor gamma coactivator 1-alpha (PGC-1α) is a key regulator of cardiomyocyte metabolism and mitochondrial function and is down-regulated in hypoxia; however, the underlying mechanism is incompletely resolved. Using primary rat cardiomyocytes coupled with electrophoretic mobility shift and luciferase assays, we report that hypoxia impaired mitochondrial energetics and resulted in an increase in nuclear localization of the Nuclear Factor-κB (NF-κB) p65 subunit, and the association of p65 with the PGC-1α proximal promoter. Tumor necrosis factor α (TNFα), an activator of NF-κB signaling, similarly reduced PGC-1α expression and p65 binding to the PGC-1α promoter in a dose-dependent manner, and TNFα-mediated down-regulation of PGC-1α expression could be reversed by the NF-κB inhibitor parthenolide. RNA-seq analysis revealed that cardiomyocytes isolated from p65 knockout mice exhibited alterations in genes associated with chromatin remodeling. Decreased PGC-1α promoter transactivation by p65 could be partially reversed by the histone deacetylase inhibitor trichostatin A. These results implicate NF-κB signaling, and specifically p65, as a potent inhibitor of PGC-1α expression in cardiac myocyte hypoxia.

## 1. Introduction

The adult mammalian heart relies heavily on mitochondria to produce ATP, primarily from fatty acid β-oxidation. However, during stress conditions, such as cardiac hypertrophy or heart failure, a metabolic switch from mitochondrial oxidative catabolism to anaerobic glycolytic pathways is utilized to produce energy. The peroxisome proliferator-activated receptor (PPAR)-γ coactivator-1α (PGC-1α) is a key transcription factor regulating mitochondrial oxidative metabolism, biogenesis, and respiration [1]. Previous studies have shown that PGC-1α is regulated by the Nuclear Factor-κB (NF-κB) [2]. NF-κB is a central regulator of inflammatory processes, cell proliferation, tumorigenesis, cardiac hypertrophy, and cell survival [3]. The NF-κB family is comprised of five subunits that include RelA/p65, RelB, c-Rel, p100/p52, and p105/p50. The heterodimer p65/p50 is the most common form in mammalian cells including cardiac myocytes. NF-κB is stimulated in response to various biological signals, including reactive oxygen species (ROS) production, or in response to cytokines such as tumor necrosis factor α (TNFα) and interleukin 1β (IL-1β) [4]. TNFα is a pro-inflammatory cytokine that is produced and secreted by cardiac myocytes in response to ischemic cardiac injury, cardiac hypertrophy, and heart failure. High levels of TNFα have been detected in the plasma of humans and mice following myocardial infarction (MI), which is a major underlying cause of heart failure worldwide [5]. Furthermore, permanent coronary occlusion of TNFα knockout mice resulted in smaller infarct sizes, reduced levels of intercellular adhesion molecule 1 (ICAM-1), and decreased inflammation indicated by lower numbers of cardiac infiltrating neutrophils and macrophages following MI [5,6]. During MI or hypoxic stress, an increase in ROS generation from impaired mitochondrial bioenergetics and respiration triggers the peroxidation of lipids and proteins that ultimately results in the death of cardiac cells, contributing to ventricular dysfunction. Notably, NF-κB has been previously shown to be stimulated in cardiac myocytes by ROS following MI, and blocking NF-κB activation resulted in smaller infarct sizes and improved cardiac function [7]. Taken together these studies demonstrate that NF-κB activation during ischemic injury can be detrimental to cardiac cell survival [3,8,9].

Interestingly, the p65 subunit of NF-κB was previously shown to interact with and impair PGC-1α activity, highlighting a possible role for NF-κB in the regulation of PGC-1α [2]. This view is substantiated by a recent study in which PGC-1α expression was markedly reduced in a mouse model in which TNFα was constitutively expressed in cardiac myocytes [10]. While the study demonstrated that TNFα over-expression suppressed PGC-1α activity, the underlying mechanisms were not determined, raising the interesting possibility of a pathophysiological connection between inflammatory signaling pathways mediated by TNFα, NF-κB, and mitochondrial bioenergetic dysfunction during hypoxia.

In this report, we explore this possibility and provide novel compelling evidence that hypoxic stress is functionally linked to loss of PGC-1α expression in cardiac myocytes: the inflammatory factor NF-κB p65 subunit transcriptionally silences PGC-1α promoter activity, ostensibly by recruiting chromatin remodeling proteins such as histone deacetylases (HDAC), which may contribute to ongoing mitochondrial injury and cell death during hypoxia [10,11]. Hence, our findings reveal a novel signaling axis that potentially links mitochondrial dysfunction and inflammatory responses during hypoxic stress to a transcriptional mechanism that impinges upon PGC-1α expression.

## 2. Materials and Methods

### 2.1. Animals

All studies involving animals were approved by the Animal Care Committee of the University of Manitoba and were in accordance with the guidelines of the Canadian Council on Animal Care, directive 2010/63/EU, and the National Institutes of Health (NIH). Animals were housed in an animal facility with standard laboratory conditions, with a 12–12 h light-dark cycle. Water and standard rodent chow (Envigo, 2914) were available ad libitum.

### 2.2. Cell Culture and Treatments

Cardiac myocyte cells were isolated from 1- to 2-day-old Sprague-Dawley rats. Animals were sacrificed by cervical dislocation, and hearts were removed and processed enzymatically to isolate cardiac myocyte cells, which were then cultured as previously reported [12]. After plating the cultured cells in serum-free DMEM for 24 h (1 pack DMEM/F12 (Thermo Fisher Scientific, Waltham, MA, USA, MT10092 CV), 3 mM NaHCO_3_, 15 mM HEPES (Sigma-Aldrich, St. Louis, MO, USA, H3375), 1 mM Na selenite (Sigma-Aldrich, St. Louis, MO, USA, S5261-100 g), 5 mg/mL transferrin (Sigma-Aldrich, St. Louis, MO, USA, T1408-500 mg), 1 mM LiCl, 125 μM ascorbic acid (Sigma-Aldrich, St. Louis, MO, USA, A4034-100 g), 4 mg/mL insulin (Sigma-Aldrich, St. Louis, MO, USA, I0516-5 mL), 1 mL T3 (L-Thyroxine-1 mL), and 1 mL/L gentamicin (Thermo Fisher Scientific, Waltham, MA, USA, 15,710,072), cells were transfected with expression plasmids using Effectene reagent (Qiagen, Inc., Hilden, Germany, 301,427), as previously described [12]. For studies involving hypoxia, cells were incubated in an air-tight chamber (95% N_2_, 5% CO_2_, pO_2_ ≤ 5 mm Hg) for 12 h.

### 2.3. Basic RNA-seq Data Processing

Cardiac-specific conditional NF-κB p65 KO mice were created by crossing p65 floxed mice (p65^fl/fl^) with mice in which Cre recombinase is expressed under the control of the α-myosin heavy chain (MHC) promoter driven by Mer-Cre-Mer (α-MHC-MCM). Mice (α-MHC-MCM; p65^fl/fl^) received tamoxifen for 5 days (oral gavage) to generate NF-κB p65 KO, and were sacrificed within a week following the last dose of tamoxifen [13]. Wild-type or p65 KO mice were anesthetized, followed by excision of the hearts. Hearts were washed in phosphate-buffered saline (PBS; 8 g NaCl, 0.2 g KCl, 1.15 g Na_2_HPO_4_.7H_2_O, 0.2 g KH_2_PO_4_, pH 7.4) and kept at −80 °C until RNA extraction. Total RNA was extracted from whole hearts by crushing the heart manually in 1 mL Trizol (Invitrogen, Waltham, MA, USA, 15,596,018). After crushing, Chloroform (200 μL) (Sigma-Aldrich, St. Louis, MO, USA, B9673-200 mL) was added to the hearts and incubated for 3 min, at room temperature (RT), followed by centrifugation at 12,000× *g* for 15 min, with collection of the supernatant and addition of 500 μL cold isopropanol (Thermo Fisher Scientific, Waltham, MA, USA, A464-4). This step was followed by centrifugation at 12,000× *g* for 10 min. The RNA pellet was washed in 500 μL cold 75% ethanol, followed by centrifugation at 7500× *g* for 5 min. After washing twice, the RNA pellet was placed on ice and air dried. The dry pellet was reconstituted by the addition of DNase-free water (Thermo Fisher Scientific, Waltham, MA, USA, AM9937). RNA-seq was performed by McGill University and the Génome Québec Innovation Center, Canada, as previously described [14]. In brief, paired-ends sequences were clipped for adapters, followed by trimming for minimum quality (Q30) in 3′ and filtering for a minimum length of 32 bp with Trimmomatic version 0.36. The remaining read pairs were aligned to the ENSEMBL Mus Musculus assembly (release 83) using STAR version 2.5.3a two-pass approach [15]. Gene-level count-based quantification against ENSEMBL gene annotations was carried out with HT-seq count in the intersection-non-empty mode [16]. The RNA-seq process was completed through the GenPipes framework [16].

### 2.4. Gene Expression Analysis

Exploratory analysis was performed using different functions and packages from R and Bioconductor [17]. Differential expression was analyzed with DESeq2 [18]. Gene Ontology enriched terms were identified with gprofiler2 [19].

### 2.5. Reactive Oxygen Species Assay

Cardiac myocytes were treated with 2.5 μM dihydroethidium (Invitrogen, Waltham, MA, USA, D23107) (30 min) and imaged by Olympus AX-70 epifluorescence microscope. Enhanced red fluorescence staining indicates enhanced the production of superoxide species.

### 2.6. Mitochondrial Membrane Potential Assay

Cardiac myocytes were treated with tetra-methylrhodamine methylester perchlorate (TMRM; Thermo Fisher Scientific, Waltham, MA, USA, T-668) (50 nM) and imaged by Olympus AX-70 epifluorescence microscope. The fluorescent intensity of cells was analyzed using ImageJ. Mitochondria showing standard fluorescence intensity are visualized as bright red; dispersed mitochondrial membrane potential is visualized as very light red.

### 2.7. Mitochondrial Respiration Assay

Cardiac myocytes were incubated in XFe96 Cell Mito Stress Analyzer designated 96-well plates (Agilent, Santa Clara, CA, USA) and subjected to 12 h hypoxia, or left in normoxic conditions for control. Mitochondrial oxygen consumption rate (OCR) analysis was performed on both plates on the same day. For the OCR analysis procedure, the cell culture medium was changed 1 h prior to the assay to XF Base medium (Agilent, Santa Clara, CA, USA, 102,353–100; pH 7.4) supplemented with 1 mM pyruvate (Thermo Fisher Scientific, Waltham, MA, USA, 11,360–070) and 10 mM D-glucose (Thermo Fisher Scientific, Waltham, MA, USA, D16-500). After 1 h incubation with the XF Base medium, cells were treated with oligomycin (1 μM; Sigma-Aldrich, St. Louis, MO, USA, O4876-5 mg), FCCP (2-[2-(4-(trifluoromethoxy)phenyl)hydrazinylidene]-propanedinitrile) (1 μM; Sigma-Aldrich, St. Louis, MO, USA, C2920) and rotenone (1 μM; Sigma-Aldrich, St. Louis, MO, USA, R8875-1 g) combined with antimycin A (1 μM; Sigma-Aldrich, St. Louis, MO, USA, A8674-25 mg). The treatments were added sequentially through ports in the Seahorse Flux Pak cartridges, as reported previously [12].

### 2.8. Cell Viability Assay

Cardiac myocytes were treated with the vital dyes calcein acetoxymethylester (Calcein-AM, 2 μM; Life Technologies, Waltham, MA, USA, C3100MP) to detect live cells (visualized green) and ethidium homodimer-1 (2 μM) to detect dead cells (visualized red) by Olympus AX-70 epifluorescence microscope at ×200 magnification.

### 2.9. Luciferase Assay and Mutagenesis

Cardiac myocytes were transfected with a mouse PGC-1α luciferase reporter construct PGC1αluc, as previously reported [20,21,22]. To normalize differences in transfection efficiency, co-transfection with β-galactosidase or Renilla expression vector (pRL, 5 ng; Promega, Madison, WI, USA) was used. Data are expressed as relative light units or normalized to controls using a Dual Luciferase Reporter Assay System in a GloMax MultiPlus Luminometer (Promega, Madison, WI, USA). To mutate the M2 p65 binding site, a QuikChange XL Site-Directed Mutagenesis Kit (Agilent, Santa Clara, CA, USA) was used, with custom primers (Table 1) per the manufacturer’s directions.

### 2.10. qPCR

Cardiac myocytes were processed with GeneJet RNA Purification Kit (Thermo Fisher Scientific, Waltham, MA, USA) for extraction of total RNA. In total, 25 ng of extracted total RNA was used for all reactions and processed using a qScript One Step qRT PCR Kit (Quanta Biosciences, Beverly, MA, USA). Bio-Rad iQ5 real-time PCR thermocycler was used for amplification and analysis, with normalization to Gapdh, and relative gene expression calculated (2^−^^ΔΔCT^ method). Primer sequences were PGC-1α-forward 5′-AAGTGTGGAACTCTCTGGAACTG-3′, PGC-1α-reverse 5′-GGGTTATCTTGGTTGGCTTTATG-3′, Gapdh-forward 5′-TGCACCACCAACTGCTTAGC-3′, and Gapdh-reverse 5′-GGCATGGACTGTGGTCATGAG-3′.

### 2.11. Western Blotting

Total protein was lysed from cardiac myocytes with RIPA Lysis Buffer containing 1 mM PMSF, 0.1 mM DTT, and 1x protease inhibitor (Thermo Fisher Scientific, Waltham, MA, USA). Lysates were centrifuged at 14,000 rpm at 4 °C for 15 min. Nuclear and cytoplasmic proteins were isolated by NE-PER Nucleic and Cytoplasmic Extraction Kit (Pierce, Waltham, MA, USA). Proteins were separated on 12% resolving polyacrylamide gels and transferred to PVDF, with blocking of blots using 5% milk in PBST (0.1% Tween) for 1 h, and proteins identified using the following antibodies for 1 to 3 h: p65 (1:500; Millipore, Burlington, MA, USA), PGC-1α (1:1000; Calbiochem, St. Louis, MO, USA,) α-tubulin (1:5000; DSHB, Iowa City, IA, USA). After incubation with the primary antibodies, secondary goat anti-mouse antibodies conjugated to horse radish peroxidase were applied for 1 h. The Immuno Cruz Western Blotting Luminol Kit (Santa Cruz, Dallas, MT, USA) was used for visualization, with intensity analyzed by Bio-Rad Quantity One software and normalized to α-tubulin.

### 2.12. Electrophoretic Mobility Shift Assay

Electrophoretic mobility shift assays (EMSA) employed biotin-labelled or non-labelled (cold) oligonucleotide probes (Integrated DNA Technologies, Coralville, IA, USA) with sequences shown in Table 1. EMSA experiments were performed as singletons. COS7 cells were untreated, transfected for 24 h with pCMX empty vector or p65-pCMX vector, or treated for 4 h with varying concentrations of recombinant rat TNFα (R&D Systems, Minneapolis, MN, USA). Nuclear protein lysates were obtained with the NE-PER Nucleic Acid and Cytoplasmic Extraction Kit per the manufacturer’s directions (Pierce, Waltham, MA, USA). Lightshift EMSA Optimization and Control Kit (Thermo Fisher Scientific, Waltham, MA, USA) was used for control, shift, cold competition, and super-shift reactions, with 1 μL 50% glycerol, 100 mM MgCl_2_, and NP-40 included for all EMSA samples. NF-κB p65 antibody (Pierce, Waltham, MA, USA) was used for super-shifts. Samples were run on 6% resolving acrylamide gels and transferred to 0.45 μm Biodyne nylon membranes (Pall Life Sciences, Portsmouth, UK), cross-linked, and visualized (Chemiluminescent Nucleic Acid Detection Kit (Thermo Fisher Scientific, Waltham, MA, USA)).

### 2.13. Statistical Analysis

Data are reported as mean ± standard error for at least three independent biological replicates. Results were analyzed by a two-tailed Student’s t-test or a one-way or two-way analysis of variance (ANOVA), with Tukey post hoc analysis as appropriate, using Origin 9 (OriginLab formerly MicroCal Software, Northampton, MA, USA) or Microsoft Excel with *p* < 0.05 considered to be statistically significant. In experiments where multiple experimental groups were compared to a fixed control value, one sample t-test was performed to compare treatment groups to control, followed by pairwise t-test to compare the treatment groups.

## 3. Results

### 3.1. Mitochondrial Function, Respiration, and Cell Viability Are Impaired during Hypoxia

**Figure 1:**
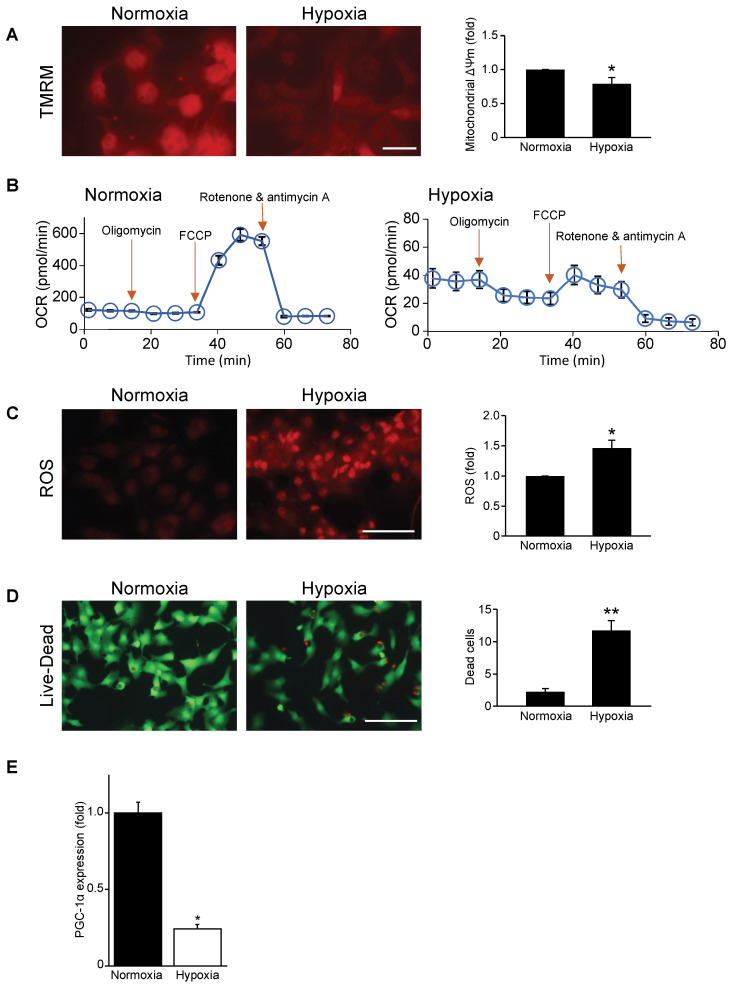
Mitochondrial function, respiration, and cell viability are impaired in hypoxic cardiac myocytes. (**A**) Mitochondrial membrane potential (∆Ψ m) during normoxia and hypoxia as assessed by tetra-methylrhodamine methylester perchlorate (TMRM) staining (red fluorescence); bar: 20 μm. (**B**) Mitochondrial oxygen consumption rate (OCR) in neonatal rat cardiac myocytes subjected to normoxia (left panel) and hypoxia (right panel) from 6 to 8 wells per condition and from 3 to 4 independent myocyte isolations. (**C**) Mitochondrial reactive oxygen species (ROS) during normoxia and hypoxia as assessed by dihydroethidium staining (red fluorescence); bar: 100 μm. (**D**) Cell viability during normoxia and hypoxia. Green cells indicate live cells and red cells indicate dead cells; bar: 100 μm. (**E**) PGC-1α mRNA expression under normoxic and hypoxic conditions; mRNA levels were normalized to Gapdh. Data are expressed as mean ± SEM. * p < 0.05 or ** p < 0.01 versus normoxia, n = 3–4 independent myocyte isolations.

Earlier studies by our group determined that mitochondrial function was impaired in cardiac myocytes during hypoxic stress [12]; however, the underlying mechanisms remained undetermined. To begin to address the underlying mechanism for this phenomenon, we validated mitochondrial injury and cell viability by subjecting cardiac myocytes to hypoxic stress. As shown by epifluorescence microscopy, in contrast to normoxic control cells, a marked decrease in mitochondrial membrane potential (∆Ψm) was observed in cardiac myocytes subjected to hypoxia (Figure 1A), coinciding with a dramatic decrease in mitochondrial OCR (Figure 1B). Notably, mitochondrial ROS production was increased in cardiac myocytes subjected to hypoxia compared to normoxic controls (Figure 1C). Moreover, impaired mitochondrial function resulted in a significant decrease in cell viability in cardiac myocytes subjected to hypoxia (Figure 1D). These findings confirm that hypoxia causes mitochondrial injury and cell death of cardiac myocytes.

### 3.2. Mitochondrial Biogenesis Factor PGC-1α Is Repressed in Hypoxic Cardiac Myocytes

Since PGC-1α is a long-term regulator mitochondrial respiration and biogenesis, we tested whether the impaired mitochondrial function of cardiac myocytes during hypoxia may be related to altered PGC-1α expression. We therefore examined PGC-1α expression in cardiac myocytes during hypoxia. As shown by quantitative real-time PCR, a significant reduction in PGC-1α mRNA abundance was detected in cardiac myocytes during hypoxia compared to normoxia (Figure 1E).

### 3.3. Hypoxia Increases Nuclear NF-κB p65 and PGC-1α Promoter Binding

**Figure 2:**
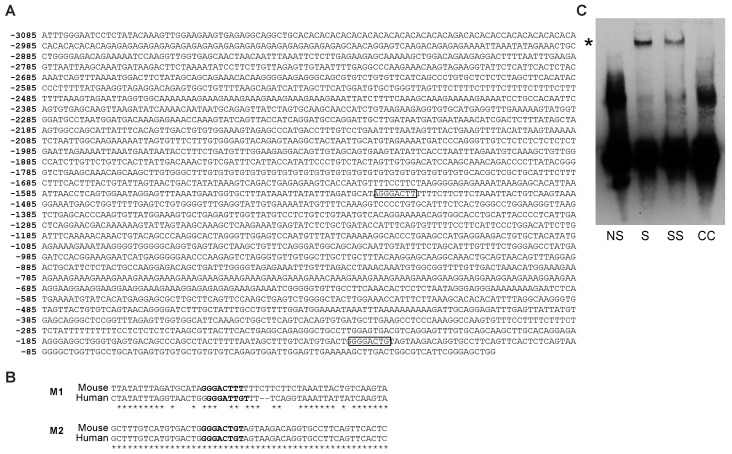
The PGC-1α gene promoter. (**A**) NF-κB p65 binding sites (boxes) in the 3 kb proximal mouse PGC-1α promoter are indicated. The binding site at -1522 is denoted as M1, while the site at −127 is M2. Numbers denote location in nucleotides relevant to the PGC-1α ATG start codon. (**B**) The region flanking the putative M1 and M2 p65 binding sites (boldface) is depicted; the M2 site exhibits complete conservation between the mouse and human PGC-1α gene promoters, whereas the M1 site is relatively poorly conserved (<70%); * denotes nucleotide identity. (**C**) Electrophoretic mobility shift assay for NF-κB p65 binding to the PGC-1α promoter. Asterisk (*) denotes shifted protein–oligonucleotide complex. NS, non-shifted control lacking cell lysate; S, shifted sample containing p65-transfected cell lysate; SS, super-shift containing p65-transfected cell lysate and anti-p65 antibody; CC, lysate including cold competition (200x unlabeled oligonucleotides).

Sequence analysis of the mouse PGC-1α promoter identified two putative canonical cis-acting binding elements for the cellular factor NF-κB, M1, and M2 located at −1522 and −127 relative to the ATG start codon, respectively (Figure 2A) [20]. Notably, the M2 sequence and its flanking region is completely conserved in the human PGC-1α promoter (Figure 2B). In contrast, the mouse M1 site and flanking region is relatively poorly conserved in the human genome. Electrophoretic mobility shift assay (EMSA) confirmed that p65 bound to the wild-type PGC-1α promoter, as a shifted protein-oligonucleotide complex was only observed following p65 transfection, the intensity of this complex was notably reduced by p65 antibody super-shift, and the complex could be competed by cold (unlabeled) oligonucleotide (Figure 2C).

**Figure 3:**
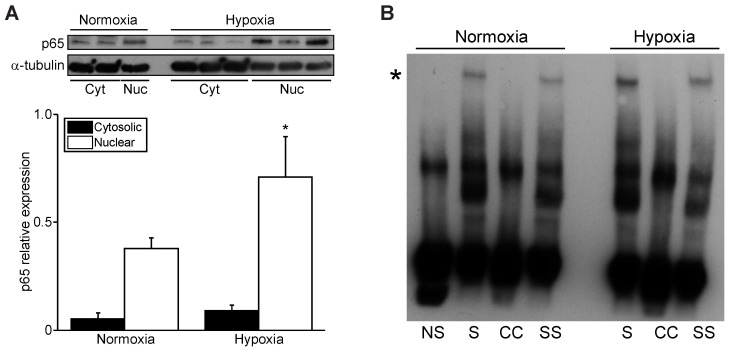
Hypoxia increases nuclear NF-κB p65 activity. (**A**) Western blot analysis of cytosolic and nuclear fractions for NF-κB p65 subunit in cardiac myocytes during normoxia or hypoxia. p65 expression is normalized to α-tubulin. * p < 0.05 versus nuclear normoxia sample. (**B**) Electromobility shift analysis of nuclear extracts for NF-κB p65 subunit binding derived from cardiac myocytes under normoxic and hypoxic conditions. Asterisk (*) denotes shifted protein–oligonucleotide complex, which is depleted following p65 antibody super-shift (SS). NS, non-shifted control lacking cell lysate; S, shifted sample containing p65-transfected cell lysate; CC, shift using cold competition (200x unlabeled oligonucleotides).

Previous work by our laboratory showed that the p65 subunit of NF-κB can transcriptionally silence certain gene promoters, raising the interesting possibility that NF-κB may transcriptionally repress PGC-1α during hypoxia [23,24]. To examine this possibility, we monitored the expression levels of cytoplasmic and nuclear p65 NF-κB subunit as a surrogate for NF-κB activity in cardiac myocytes during hypoxia. As demonstrated by Western blot analysis, a marked upregulation in nuclear p65 NF-κB expression was detected in hypoxic cardiac myocytes (Figure 3A). Furthermore, this coincided with a marked increase in nuclear NF-κB DNA binding in nuclear lysates from cardiac myocytes subjected to hypoxia as demonstrated by EMSA (Figure 3B). These findings verify that hypoxia induces NF-κB p65 nuclear translocation and recruitment to the PGC-1α promoter.

### 3.4. NFκB p65 Represses PGC1α Promoter Transactivation

**Figure 4:**
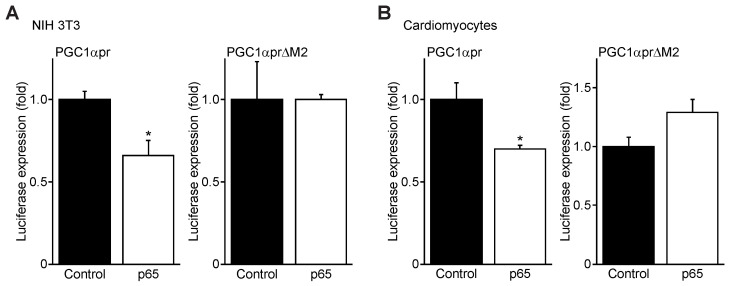
PGC-1α promoter activity is repressed by NF-κB. (**A**,**B**) Luciferase reporter assay of the mouse PGC-1α promoter in (**A**) NIH 3T3 cells or (**B**) rat post-natal cardiac myocytes transduced with empty vector alone (Control) or NF-κB p65 expression cassette, with normalization to co-transfected Renilla luciferase. * p < 0.05 versus Control.

To gain further insight into the regulation of the PGC-1α gene promoter by NF-κB, we next examined the effects of the NF-κB p65 subunit on PGC-1α promoter activation in NIH 3T3 fibroblasts and post-natal cardiac myocytes. For these studies, we generated luciferase promoter reporter constructs harboring the mouse wild-type PGC-1α promoter (PGC1αpr) and PGC-1α promoter mutants in which the proximal canonical NF-κB element had been deleted (PGC1αpr∆M2). In NIH 3T3 fibroblasts, a marked reduction in wild-type PGC1αpr luciferase reporter activity was observed in cells expressing NF-κB (Figure 4A). In contrast, however, the luciferase activity of the PGC1αpr∆M2 promoter mutant was unaffected in cells over-expressing the p65 subunit, suggesting that this binding site was solely responsible for p65-mediated PGC-1α promoter repression. Importantly, similar findings were observed in post-natal cardiac myocytes (Figure 4B). Taken together, these findings not only confirm that the PGC-1α promoter is negatively regulated by NF-κB, but also demonstrate a cell autonomous role for the negative regulation of PGC-1α by NF-κB.

### 3.5. TNFα Represses PGC-1α Expression and Induces p65 Binding to the PGC-1α Promoter M2 Site

**Figure 5:**
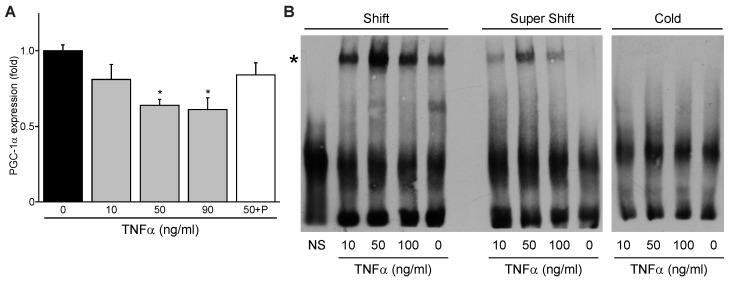
TNFα represses PGC-1α expression and increases NF-κB p65 binding to the proximal M2 site in the PGC-1α promoter. (**A**) Dose-dependent repression of PGC-1α mRNA expression by TNFα, with attenuation by parthenolide, in post-natal rat cardiac myocytes. * p < 0.05 versus 0 TNFα treatment. (**B**) Electrophoretic mobility shift assay for p65 binding to the PGC-1α promoter proximal M2 site in response to TNFα. *, shifted protein–oligonucleotide complex. NS, non-shifted control lacking cell lysate.

Since NF-κB is known to be activated classically by inflammatory cytokines including TNFα during ischemic and/or hypoxic stress, we next tested whether TNFα influences PGC-1α expression. A dose-dependent decrease in PCG-1α mRNA expression was observed in cardiac myocytes treated with TNFα, peaking at 50 ng/mL TNFα (Figure 5A). This repression could be attenuated by the p65 inhibitor parthenolide. The decline in PGC-1α expression was accompanied with a corresponding increase in p65 NF-κB binding to the PGC-1α promoter M2 site as shown by EMSA, similarly peaking with 50 ng/mL TNFα treatment (Figure 5B). These findings demonstrate that TNFα increases NF-κB p65 binding to the PGC-1α promoter and suppresses PGC-1α expression.

### 3.6. Bioinformatics of Cardiac-Restricted p65 Gene Knockout in Mice

**Figure 6:**
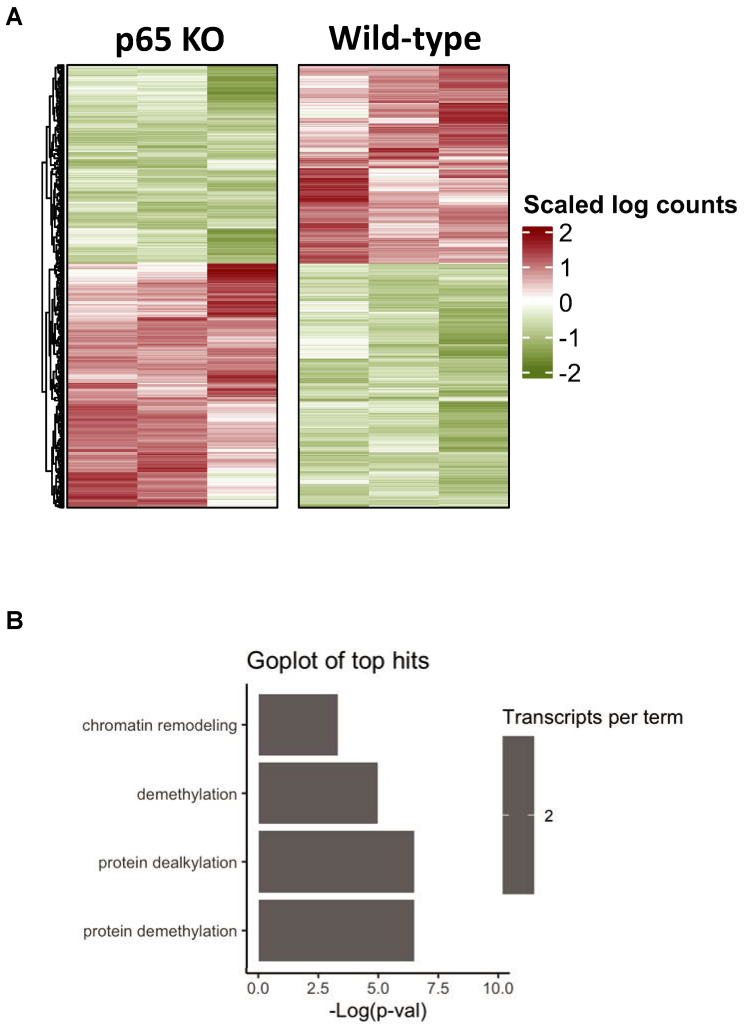
RNA-seq analysis of wild-type and cardiac-specific p65 knockout mice. (**A**) Heat map analysis of NF-κB p65 knockout mice (p65 KO) and wild type mice. (**B**) Top gene ontology terms for genes altered in p65 KO mice compared to wild-type mice.

To begin to explore the underlying mechanism by which NF-κB suppresses PGC-1α activity, we used a bioinformatics approach and RNA-seq analysis to assess baseline changes in wild-type mice hearts and mice in which the p65 NF-κB subunit was ablated in a cardiac-restricted manner (p65 KO). Heat map analysis revealed significant changes in gene expression levels between wild-type and p65 KO mice (Figure 6A). Interestingly, gene ontology (GO) analysis identified chromatin remodeling as one of the top cellular processes that was different between wild-type and p65 KO mice hearts (Figure 6B).

### 3.7. PGC-1α Promoter Is Repressed by NF-κB and Chromatin Remodeling Histone Deacetylases

**Figure 7:**
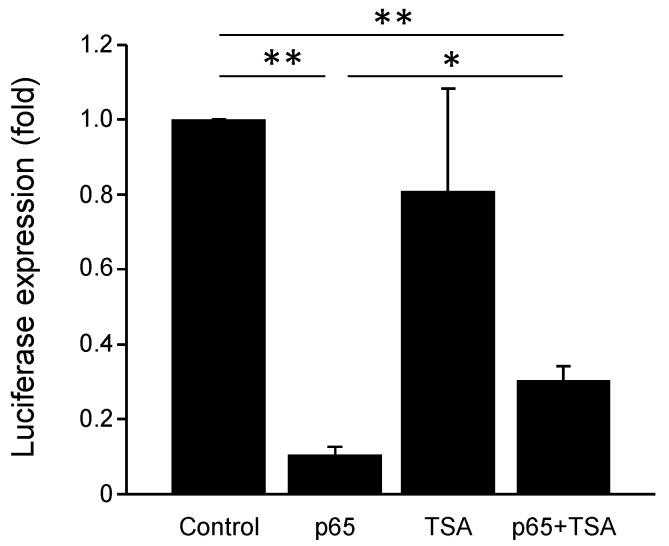
Luciferase reporter assay for the PGC-1α promoter. Cardiac myocytes were transduced with empty vector (Control) or a vector encoding NF-κB p65 subunit and PGC-1α promoter reporter and monitored for PGC-1α promoter activity in the absence and presence of trichostatin A (TSA, 10 μM). TSA alone did not affect PGC-1α promotor activity, whereas TSA in the presence of NF-κB p65 vector partially reversed the decrease in PGC-1α promotor activity by p65. * p < 0.05 or ** p < 0.01 versus indicated sample.

We tested the possibility that the inhibitory effects of NF-κB on PGC-1α promoter activity were related to influences of chromatin remodeling proteins, specifically histone deacetylases (HDACs), which were previously shown to be important for suppressing gene expression in cardiac myocytes [11,23]. We previously reported that HDAC5 represses PGC-1α expression, and that hypoxia up to 12 h decreases histone H3 acetylation at K9 in cardiac myocytes [20,21]. We therefore transduced post-natal cardiac myocytes with the PGC-1α promoter luciferase reporter with or without an expression vector for NF-κB p65, and in the absence or presence of the HDAC inhibitor trichostatin A (TSA). PGC-1α promoter activity was significantly suppressed in cells expressing p65 NF-κB in the absence of TSA; however, this inhibitory effect was significantly abrogated by the presence of TSA (Figure 7). These findings indicate that NF-κB represses PGC-1α promoter transactivation through a mechanism that involves chromatin remodeling by HDAC proteins.

## 4. Discussion

Previous studies have shown that mitochondrial function and oxidative metabolism are impaired in the heart during ischemic/hypoxic stress injury; however, the underlying mechanisms remain cryptic [25]. The transcriptional coactivator PGC-1α was previously shown to play a fundamental role in the transcriptional regulation of cardiac energy metabolism. PGC-1α regulates genes encoding enzymes involved in cardiac glucose and fatty acid metabolism, as well as mitochondrial biogenesis. Therefore, impaired activity of PGC-1α may contribute to mitochondrial perturbations and consequent cardiac cell viability during cardiac ischemic injury [26]. Herein, we provide new compelling evidence that PGC-1α is transcriptionally silenced by the innate immunity factor NF-κB. We specifically showed that impaired mitochondrial bioenergetics coincided with a marked decrease in PGC-1α mRNA abundance in cardiac myocytes subjected to hypoxia, leading us to reason that hypoxia-induced mitochondrial perturbations may be related in part to impaired PGC-1α activity. While we did not assess PGC-1α protein in the present study, we have previously demonstrated that long-term hypoxia in vivo results in significant down-regulation of PGC-1α protein in both the left and right ventricles of the heart; thus, our present data provide a potential mechanism for ongoing mitochondrial dysfunction in the setting of chronic hypoxia [21].

NF-κB is a key player in the crosstalk between inflammation and ischemic/hypoxic stress injury. During myocardial infarction, various pro-inflammatory cytokines including TNFα, IL-6, IL-1β, and TGF-β_1_ are released, promoting cardiac remodeling [27]. TNFα specifically triggers production of mitochondrial ROS in cardiac myocytes, which may lead to mitochondrial DNA damage and development of heart failure [28]. TNFα is also a classical activator of innate immune signaling through NF-κB. Notably, sequence analysis of the mouse PGC-1 promoter identified the presence of highly conserved canonical cis-acting DNA binding sites for the p65 NF-κB subunit, raising the possibility that PGC-1α expression may be regulated by NF-κB. Concordant with this view, we observed a marked increase in NF-κB p65 subunit nuclear localization and PGC-1α promoter DNA binding in cardiac myocytes during hypoxia. These findings support the notion that PGC-1α gene promoter activity may be directly regulated by NF-κB. Indeed, over-expression of the NF-κB p65 subunit repressed PGC-1α promoter activity, while mutation of the PGC-1α promoter proximal M2 binding element attenuated the inhibitory effects of NF-κB. Using two independent approaches, we showed that PGC-1α promoter activity was repressed by NF-κB: Over-expression of NF-κB p65 subunit repressed PGC-1α promoter activity in cardiac myocytes and fibroblasts, indicating a cell autonomous effect of PGC-1α regulation by NF-κB. The inflammatory cytokine TNFα repressed PGC-1α expression, recapitulating the effects of hypoxia, and this effect was blocked by the p65 inhibitor parthenolide.

Another novel feature of our study was the bioinformatic analysis of the wild-type and cardiac-specific p65 knockout mice, which revealed that the top regulated GO pathway was chromatin remodeling. This finding suggests that chromatin remodeling proteins may be involved in the negative regulation of PGC-1α promoter activity. Indeed, earlier work by our group and others has shown that, in addition to NF-κB’s ascribed role as a transcriptional activator, it can also repress the activity of certain promoters through the recruitment of chromatin modifying proteins such as histone deacetylases (HDACs). In particular, the class I HDACS (HDAC1, HDAC2, HDAC3, and HDAC8) can form protein interactions with NF-κB and suppress target gene promoter activity, and have also been shown to regulate vital processes in cardiac myocytes including cell growth and viability [11,23,29]. Our finding that trichostatin A, a broad inhibitor of HDACs, attenuated the inhibitory effects of NF-κB on PGC-1α promoter activity suggests that chromatin modifying HDAC proteins are involved in the transcriptional silencing of PGC-1α by NF-κB [29]. This finding agrees with our previous report that 12 h hypoxia in cardiac myocytes results in deacetylation of histone H3 at the K9 residue, and that TSA attenuated hypoxia-dependent loss of PGC-1α mRNA expression [21]. In this regard, further investigations are required to establish which specific HDAC is recruited to the PGC-1α promoter during hypoxia, and whether other chromatin modifications are operationally involved in PGC-1α regulation in cardiac myocytes.

Based on the current findings, we propose a model in which NF-κB activation during hypoxia or downstream of TNFα disrupts mitochondrial bioenergetics and mitochondrial function through a mechanism that impinges upon the transcriptional repression of PGC-1α gene activity. Hence, our findings provide the first evidence that mitochondrial dysfunction observed during hypoxia may be related to impaired PGC-1α activity through a mechanism that involves NF-κB. Interventions that modulate TNFα-NF-κB signaling may prove to be beneficial in preserving PGC-1α activity and mitochondrial integrity during ischemic or hypoxic stress.

## Figures and Tables

**Table 1:** Primers used for EMSA and mutagenesis (Mut.). The p65 binding site is underlined, and mutated nucleotides are in lowercase.

Primer	Sequence	Use
PGC1a-M2-fwd	5′-GCTTTGTCATGTGACTGGGGACTGTAGTAAGACAGGTGCCTTCAG-3′	EMSA
PGC1a-M2-rev	5′-CTGAAGGCACCTGTCTTACTACAGTCCCCAGTCACATGACAAAGC-3′	EMSA
PGC1a-∆M2-fwd	5′-GCTTTGTCATGTGACTGGaGctcGTAGTAAGACAGGTGCCTTCAG-3′	Mut.
PGC1a-∆M2-rev	5′-CTGAAGGCACCTGTCTTACTACgagCtCCAGTCACATGACAAAGC-3′	Mut.

## Data Availability

The data presented in this study are available on request from the corresponding author.
